# Temporal trends in mortality involving atrial fibrillation and rheumatic heart disease: a 25-year nationwide analysis

**DOI:** 10.3389/fcvm.2025.1687555

**Published:** 2025-12-12

**Authors:** Mohamed Fawzi Hemida, Maryam Saghir, Alyaa Ahmed Ibrahim, Anika Goel, Amna Amir Jalal, Krish Patel, Eshal Saghir, Mirna Hussein, Abdrabo Gamal, Mustafa Alsaadi, Maha Sajjad, Mahmoud Tablawy, Omar Alkasabrah, Mohammed Hammad Jaber Amin

**Affiliations:** 1 Faculty of Medicine, Alexandria University, Alexandria, Egypt; 2 Department of Medicine, Jinnah Sindh Medical University, Karachi, Pakistan; 3 Department of Medicine, Kakatiya Medical College, Warangal, India; 4 Department of Medicine, C.U. Shah Medical College, Surendranagar, India; 5 Department of Medicine, Dow University of Health Sciences, Karachi, Pakistan; 6 Faculty of Medicine, Menoufia University, Menoufia, Egypt; 7 Department of Medicine, Jabir Ibn Hayyan Medical University, Kufa, Iraq; 8 Department of Medicine, King Edward Medical University, Lahore, Pakistan; 9 Faculty of Medicine, Al-Azhar University, Cairo, Egypt; 10 Department of Internal Medicine, New York Medical College/Landmark Medical Center, Woonsocket, RI, United States; 11 Department of Medicine, Al Zaiem Al Azhari University, Khartoum, Sudan

**Keywords:** atrial fibrillation, rheumatic heart disease, trends, mortality, joinpoint regression analysis

## Abstract

**Background:**

Atrial fibrillation (AF) and rheumatic heart disease (RHD) can coexist with potential for serious complications. Trends involving both conditions remain unexplored and this study aims to explore them.

**Methods:**

Nationwide mortality records were obtained from the Centers for Disease Control and Prevention Wide-Ranging Online Data for Epidemiologic Research (CDC-WONDER) database from 1999 to 2023 among U.S. adults >45 years with AF (ICD-10 code: I48) and RHD (I05-I09). Age-adjusted mortality rates (AAMRs) were calculated per 100,000 population and stratified by demographic variables. Joinpoint regression analysis was used to determine the average and annual percent change (AAPC and APC).

**Results:**

From 1999 to 2023, a total of 36,701 deaths were reported among individuals aged >45 years with AF and RHD in the U.S. The AAMR increased from 1.04 in 1999 to 2.00 in 2023 (AAPC: 2.78; *p* *=* 0.001). Women had higher overall AAMR (1.34) (AAPC: 2.48; *p* < 0.001) than men (1.06) (AAPC: 4.14; *p* < 0.001). Racially, the highest overall AAMR was in Non-Hispanic (NH) White (1.32) while the overall AAMR in Hispanics was (0.73). Regionally, the highest overall AAMR was noticed in the West (1.68), followed by the Midwest (1.36). The majority of deaths occurred in inpatient medical facilities (13,939 deaths, 38%). Rural areas had higher overall AAMR (1.2) compared to urban areas (1.1).

**Conclusion:**

Trends in AF and RHD mortality increased lately. Higher trends observed in women, rural areas, the West region, NH white population and inpatient medical facilities.

## 1 Introduction

Rheumatic heart disease (RHD), a worldwide health issue, is viewed to be a significant cause of morbidity and mortality (1, 2). RHD develops as a complication of acute rheumatic fever that occurs after a throat infection due to group A beta-hemolytic streptococci infection. In individuals with genetic predisposition, an autoimmune reaction occurs against connective tissues and organs, particularly the heart, causing permanent valve injury and cardiac failure (2, 3). Globally, it affects more than 40.5 million individuals, resulting in more than 300,000 deaths annually, and is considered a significant public health issue. In 2015, the worldwide prevalence of RHD was approximately 34 million cases, with around 320,000 deaths related to RHD (2, 4). A study regarding demographics and mortality trends of valvular heart diseases (VHDs) in older adults in the US demonstrated that there was an increase in age adjusted mortality rates (AAMRs) for rheumatic VHDs (APC: 5.01 [95% CI: 2.01–6.92]) from 2017 to 2019 regarding previous years (5).

Atrial fibrillation (AF) is a highly prevalent cardiac problem characterized by cardiac dysrhythmia and lack of coordinated contractions, enhancing blood coagulation, clot formation, and embolic stroke (6). AF affects millions worldwide and increases the risk of stroke by fivefold, considered the most frequently diagnosed dysrhythmia in clinical practice (7). Several risk factors increase the risk of AF, such as hypertension, diabetes, obesity, age, valvular heart disease, and cardiomyopathy (8). Historically, RHD was recognized as an important etiological contributor to AF, particularly in younger populations and in regions with high RHD prevalence (9, 10). AF is expected to affect 6–12 million individuals in the USA by 2050 and 17.9 million in Europe by 2060. AF utilizes significant health resources globally, considering it a public health challenge with increased mortality risk (9).

Patients with RHD, particularly mitral stenosis, have an increased risk of developing AF. A study regarding the prevalence and predictors of atrial fibrillation in rheumatic valvular heart disease demonstrates that chronic AF is detected in 29% of 250 patients with pure mitral stenosis (10). Regarding this association, AF and RHD can coexist with the potential for serious complications, demonstrating that the onset of AF in RHD patients is a clinical marker of bad outcomes and is associated with greater morbidity and mortality as compared with the non-rheumatic population (11, 12). Although AF and RHD frequently coexist with significant morbidity and mortality, few studies have examined long-term temporal trends describing how the association between AF and RHD has evolved over recent decades.

This study aims to analyze the temporal trends in atrial fibrillation associated with rheumatic heart disease over a period of 25 years. This study obtained data from the Centers for Disease Control and Prevention Wide-Ranging Online Data for Epidemiologic Research (CDC-WONDER) database from 1999 to 2023 to evaluate temporal trends in mortality of AF associated with RHD among US adults aged over 45 years. Ultimately, this analysis seeks to provide an essential tool for identifying high-risk populations to reduce AF associated RHD related death.

## 2 Methods

### 2.1 Study setting and population

In this population-based retrospective mortality trend analysis, we conducted an analysis using death certificate data retrieved from the CDC WONDER database and analyzed data for adults aged 45 and older between 1999 and 2023 to examine mortality trends pertaining AF associated with RHD. Diagnostic coding was employed using the International Statistical Classification of Diseases and Related Health Problems-10th Revision (ICD-10) as follows: I48 for AF and I05–I09 for RHD. These ICD codes have been previously used to identify Atrial Fibrillation and RHD in administrative databases (13–15). We utilized the Multiple Cause of Death database to identify deaths in which both AF and RHD were listed as either underlying or contributing causes. This approach captures deaths where AF and RHD coexisted, reflecting their combined mortality burden, rather than implying causality between the two conditions. Adults were defined as those who were 45 years or older at the time of death. Similar age cutoff has been used by previous studies to define older adults (16, 17). This age threshold was selected based on the reliability and completeness of data within the CDC WONDER database. Preliminary explorations indicated substantial suppression of mortality data among individuals under 45 years due to small cell counts, which could lead to unstable rate estimates. Therefore, focusing on adults aged ≥45 years allowed for consistent trend evaluation and demographic comparisons. Institutional review board approval was not required for this study as it used de-identified public use data provided by the government and adhered to the Strengthening the Reporting of Observational Studies in Epidemiology (STROBE) guidelines for reporting (18).

### 2.2 Data abstraction

Data for population size, year and demographics such as Sex, Age, Race, region and states was extracted. Place of Death was categorized into Medical Facilities, Hospice, Home and Nursing Home/Long-Term care facilities. Racial and ethnic categories were classified as non-Hispanic (NH) White, NH Black or African American, Hispanic or Latino, NH American Indian or Alaskan Native, and NH Asian or Pacific Islander. The National Center for Health Statistics Urban-Rural Classification Scheme was used to assess the population by urban (large metropolitan area [population ≥1 million], medium/small metropolitan area [population 50,000–999,999]) and rural (population <50,000) counties per the 2013 U.S. census classification (19). Regions were stratified into Northeast, Midwest, South, and West according to the U.S. Census Bureau definitions (20).

### 2.3 Statistical analysis

Crude and age adjusted mortality rates (CMRs and AAMRs) per 100,000 population from 1999 to 2023 by year, sex, race/ethnicity, state, and urban-rural status with 95% CIs were calculated, using the 2000 U.S. population as the standard (21). Crude mortality rates were determined by dividing the number of AF and RHD related deaths by the corresponding U.S. population of that year. To quantify national annual trends in Atrial Fibrillation and RHD-related mortality, the Joinpoint Regression Program (Joinpoint V 5.4.0.0, National Cancer Institute) was used to determine the annual percent change (APC) with 95% CI in AAMR (22). This method allows identification of significant changes in AAMR over time by fitting log-linear regression models where temporal variation occurred. APCs were considered increasing or decreasing if the slope describing the change in mortality was significantly different from zero using two tailed *t* testing. A value of *p* < 0.05 was considered statistically significant. This analysis is descriptive and ecological in nature; no individual-level covariate adjustment or causal inference was performed.

## 3 Results

**Figure 1:**
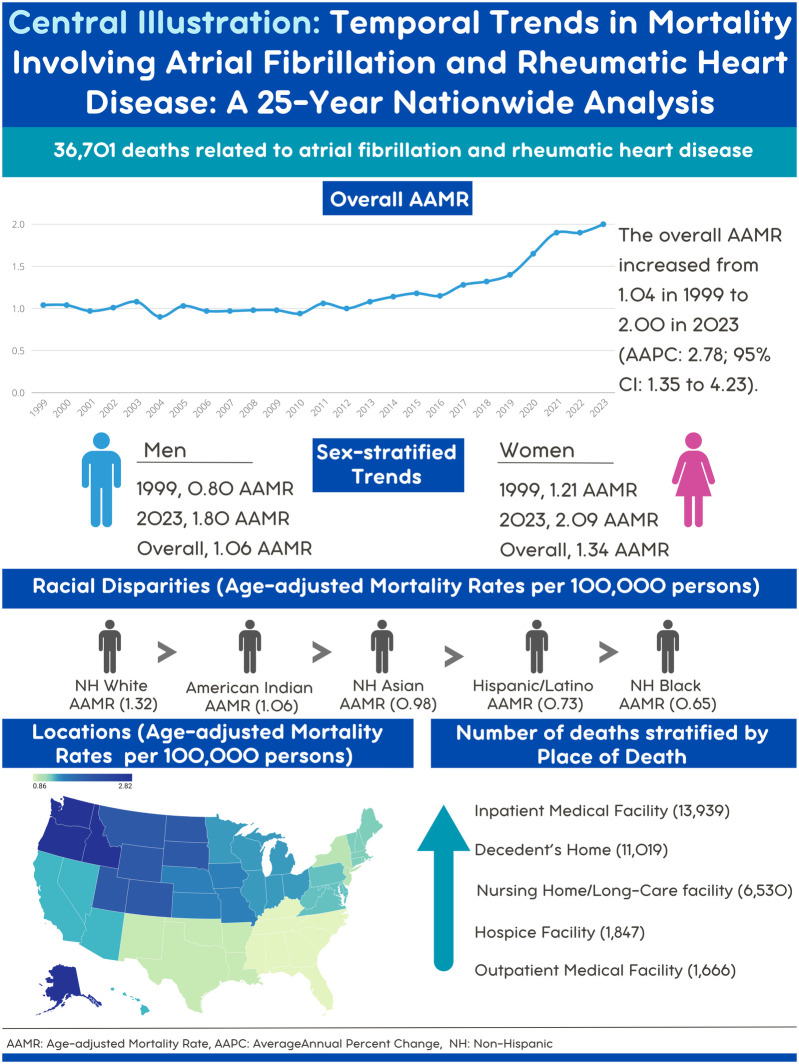
Central illustration. Map created with Datawrapper.

A total of 36,701 deaths occurred among individuals aged >45 years with AF and RHD between 1999 and 2023 in the United States. The highest number of deaths was recorded in inpatient medical facilities (13,939), followed by the decedent's home (11,019), nursing homes (6,530), hospice (1,847), and outpatient medical facilities (1,666) (Figure 1) (Supplementary Tables 1, 2).

### 3.1 Overall trends

**Figure 2:**
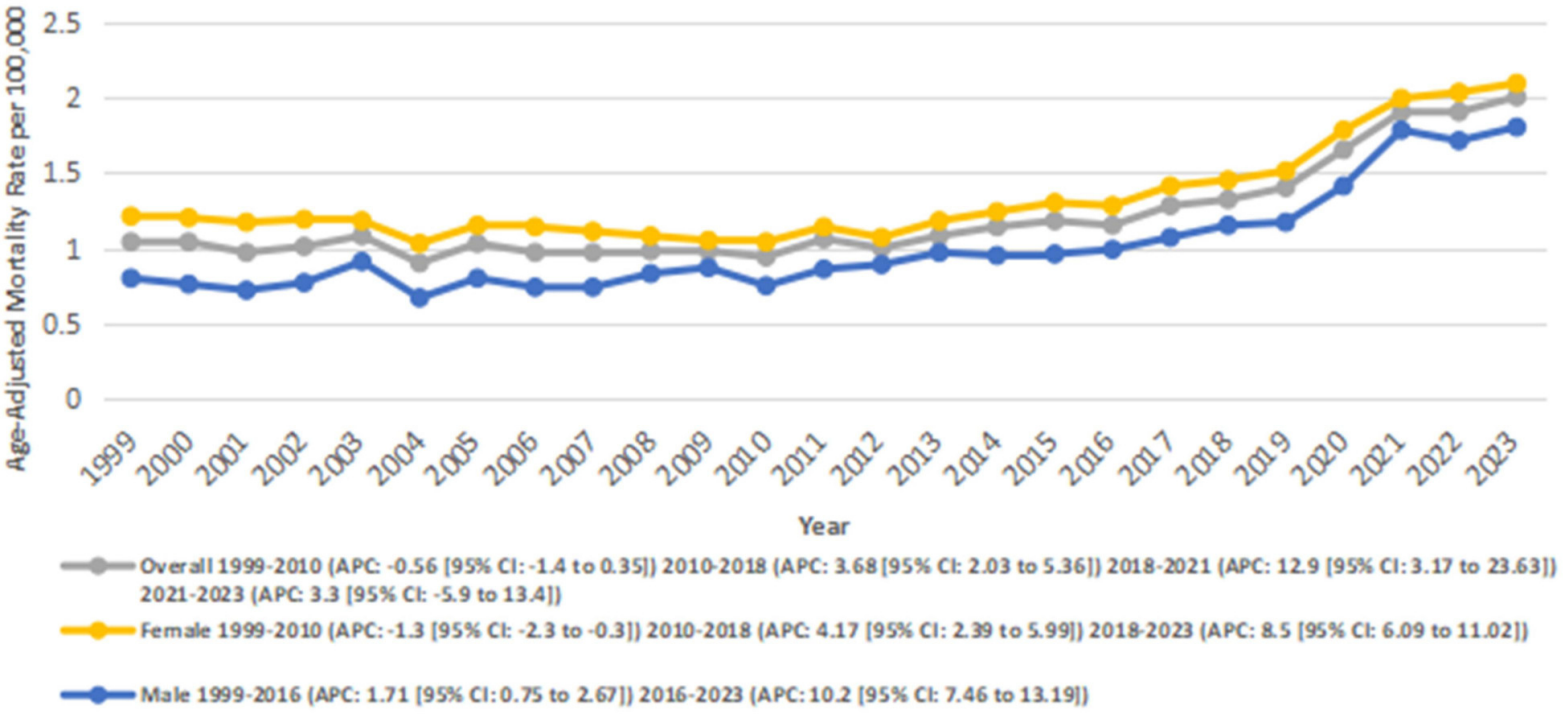
Overall and Sex-stratified atrial fibrillation and rheumatic heart disease-related AAMRs per 100,000 in adults in the United States 1999–2023.

The AAMR for AF- and RHD-related mortality in adults increased significantly from 1.04 in 1999 to 2.00 in 2023, with an AAPC of 2.78* (95% CI: 1.35–4.23; *p* = 0.001). A decrease in AAMR was observed from 1.04 in 1999 to 0.94 in 2010 (APC: −0.56; 95% CI: −1.47 to 0.36; *p* = 0.21), followed by a significant rise between 2010 and 2018 as the AAMR increased from 0.94 to 1.32 (APC: 3.69*; 95% CI: 2.04–5.37; *p* = 0.002), and from 1.32 to 1.9 between 2018 and 2021(APC: 12.95*; 95% CI: 3.18–23.64; *p* = 0.012). This was followed by a nonsignificant increase in AAMRs from 1.9 in 2021 to 2.00 in 2023 (APC: 3.31; 95% CI: −5.90 to 13.43; *p* = 0.47) (Figure 2) (Supplementary Tables 1, 3, 4).

### 3.2 Sex-stratified trends

Women had a higher overall number of deaths as compared to men (24274 vs. 12427). Women also experienced an overall higher AAMR compared to men (1.32 vs. 1.00).

In males, the AAMR increased significantly overall from 0.8 in 1999 to 1.8 in 2023 (AAPC: 4.14*; 95% CI: 3.16–5.14; *p* < 0.001). A significant increase in AAMR was observed from 0.8 to 0.99 between 1999 and 2016 (APC: 1.71*; 95% CI: 0.75–2.68; *p* = 0.0013), and from 0.99 in 2016 to 1.8 in 2023 (APC: 10.30*; 95% CI: 7.47–13.20; *p* < 0.001).

Similarly, in females, the AAMR increased significantly from 1.21 in 1999 to 2.09 in 2023 (AAPC: 2.48*; 95% CI: 1.65–3.33; *p* < 0.001). A slight decrease in AAMRs was observed from 1.21 to 1.04 between 1999 and 2010 (APC: −1.33; 95% CI: −2.35 to −0.31; *p* = 0.014), followed by a significant increase from 1.04 in 2010 to 1.45 in 2018 (APC: 4.18; 95% CI: 2.39–6.0; *p* = 0.001)., and from 1.45 to 2.09 between 2018 and 2023 (APC: 8.53*; 95% CI: 6.09–11.03; *p* = 0.001) (Figure 2) (Supplementary Tables 1, 3, 4).

### 3.3 Stratified by race

When stratified by race, the highest number of deaths was recorded for the NH White population (32,055), followed by the NH Black population (1,754), and Hispanics (1,594). NH Whites also had the highest overall AAMR (1.30), followed by the Hispanics (0.69), and NH Blacks (0.60).

Among the Hispanic or Latino population, mortality rates declined significantly from 0.71 in 1999 to 0.48 in 2013 (APC: −1.05*; 95% CI: −3.18 to 1.14; *p* = 0.33). From 2013 to 2023, mortality rates increased significantly from 0.48 to 1.1 (APC: 6.81*; 95% CI: 4.51–9.16; *p* = 0.000004). Overall, from 1999 to 2023, AAMR increased significantly from 0.71 to 1.1 (AAPC: 2.15*; 95% CI: 0.67–3.67; *p* = 0.004).

Among NH Black or African American individuals, mortality rates increased significantly from 0.47 in 1999 to 0.51 in 2016 (APC: 1.84*; 95% CI: 0.61–3.07; *p* = 0.005), and from 0.51 in 2016 to 1.2 in 2023 (APC: 12.63*; 95% CI: 9.48–15.86; *p* < 0.001). Overall, the AAMR increased significantly from 0.47 in 1999 to 1.2 in 2023 (AAPC: 4.87*; 95% CI: 3.71–6.05; *p* < 0.001).

**Figure 3:**
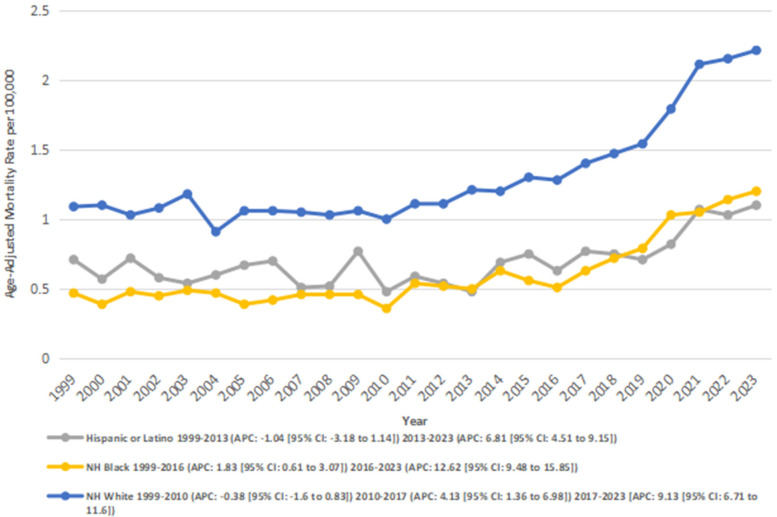
Atrial fibrillation and rheumatic heart disease-related AAMRs per 100,000 stratified by race in adults in the United States 1999–2023.

In the NH White population, a nonsignificant decline was observed from 1.09 in 1999 to 1.00 in 2010 (APC: −0.39; 95% CI: −1.6 to 0.84; *p* = 0.51), followed by a significant increase from 1.00 in 2010 to 1.4 in 2017 (APC: 4.14*; 95% CI: 1.36–7.00; *p* = 0.005), and from 1.4 in 2017 to 2.21 in 2023 (APC: 9.13*; 95% CI: 6.71–11.60; *p* < 0.001) Overall, the AAMR increased significantly from 1.09 in 1999 to 2.21 in 2023 (AAPC: 3.24*; 95% CI: −2.17 to 4.32; *p* < 0.001) (Figure 3) (Supplementary Tables 1, 3, 5).

### 3.4 Stratified by census region

AAMRs were analyzed by the U.S. Census Region to evaluate geographic variation. Differences in AAMRs and the number of deaths were observed across the four regions. The West had the highest overall AAMR (overall AAMR: 8.13; number of deaths: 118,777), followed by the Midwest (overall AAMR: 1.35; number of deaths: 9315), the Northeast (overall AAMR: 1.15; number of deaths: 7,018), and the South (overall AAMR: 0.86; number of deaths: 9,647).

In the Northeast, from 1999 to 2008, there was a significant decrease in AAMRs from 1.19 to 0.81 (APC: −4.23*; 95% CI: −6.67 to −1.72; *p* = 0.002). This was followed by a period of significant increase from 0.81 in 2008 to 1.84 in 2023 (APC: 5.37*; 95% CI: 4.32–6.43; *p* < 0.00001). Overall, the AAMRs increased significantly from 1.19 to 1.84 between 1999 and 2023 (AAPC: 1.66*; 95% CI: 0.57–2.77; *p* = 0.002).

In the Midwest, the AAMRs increased significantly from 1.04 in 1999 to 2.2 in 2023 (AAPC: 3.20*; 95% CI: 2.25–4.15; *p* < 0.001). Between 1999 and 2013, there was a nonsignificant annual increase in AAMR from 1.04 to 1.17 (APC: 0.81; 95% CI: −0.45 to 2.10; *p* = 0.20). This was followed by a significant rise in AAMR from 1.17 in 2013 to 2.2 in 2023 (APC: 6.63*; 95% CI: 4.98–8.30; *p* < 0.001).

In the South, AF- and RHD-related mortality rates decreased nonsignificantly from 0.75 in 1999 to 0.67 in 2007 (APC: −2.01; 95% CI: −4.97 to 1.04; *p* = 0.18). This was followed by a nonsignificant increase from 0.67 in 2007 to 0.76 in 2017 (APC: 2.19; 95% CI: −0.075 to 4.50; *p* = 0.06), and a significant increase from 0.76 to 1.54 between 2017 and 2023 (APC: 11,69*; 95% CI: 8.54–14.93; *p* < 0.001). Overall, between 1999 and 2023, the AAMRs increased significantly from 0.75 to 1.54 (AAPC: 3.03*; 95% CI: 1.55–4.54; *p* = 0.005).

**Figure 4:**
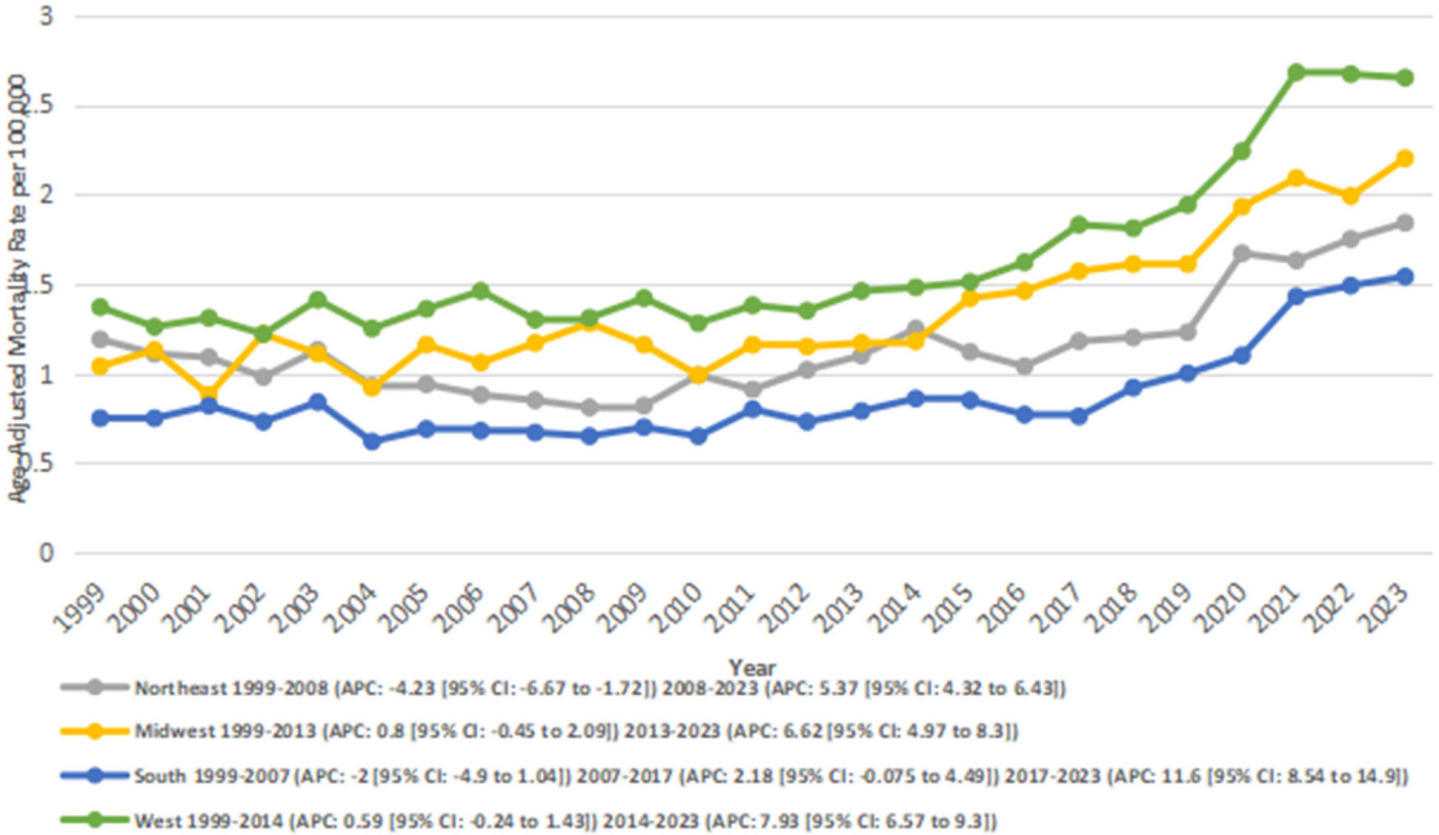
Atrial fibrillation and rheumatic heart disease-related AAMRs per 100,000 stratified by census region in adults in the United States 1999–2023.

Similarly, in the West region, an overall significant increase in AAMR was observed from 1.37 in 1999 to 2.65 in 2023 (AAPC: 3.28; 95% CI: 2.60–3.97; *p* < 0.001). In segments, AAMRs were observed to increase nonsignificantly from 1.37 in 1999 to 1.48 in 2014 (APC: 0.59; 95% CI: −0.24 to 1.44; *p* = 0.16), and this was followed by a significant increase from 1.48 in 2014 to 2.65 in 2023 (APC: 7.93*; 95% CI: 6.57–9.31; *p* < 0.001) (Figure 4) (Supplementary Tables 3, 6).

### 3.5 Stratified by urbanization

Across the study period, nonmetropolitan areas exhibited consistently higher AAMRs compared to metropolitan areas, with overall AAMRs of 1.2 and 1.1, respectively. Both metropolitan and nonmetropolitan regions experienced overall increasing trends from 1999 to 2020. In non-metropolitan areas, the AAMRs increased significantly (AAPC: 3.01*; 95% CI: 2.17–3.87; *p* < 0.001), and from 2 to 3 in metropolitan areas (AAPC: 1.51*; 95% CI: 0.68–2.34; *p* = 0.003).

According to segmental data, metro areas showed a nonsignificant decline from 7.17 in 1999 to 4.38 in 201 (APC: −1.19; 95% CI: −2.49 to 0.12; *p* = 0.072), followed by a significant increase from 4.38 in 2010 to 4.89 in 2020 (APC: 4.57*; 95% CI: 3.38–5.78; *p* < 0.001).

**Figure 5:**
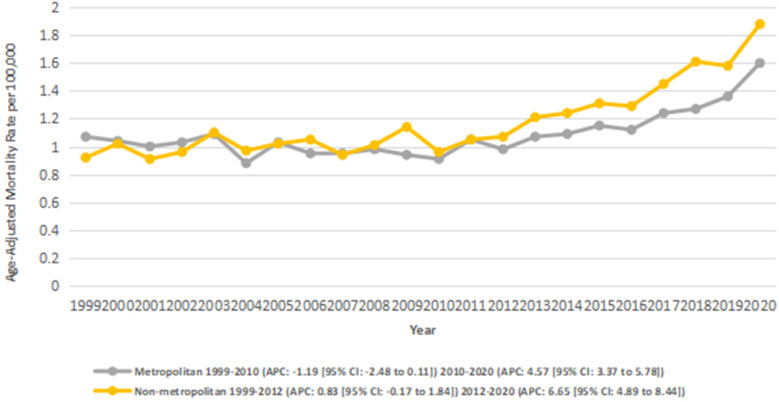
Atrial fibrillation and rheumatic heart disease-related AAMRs per 100,000 stratified by urban-rural Status in adults in the United States 1999–2020.

In non-metro areas, there was an initial non-significant incline from 7.87 in 1999 to 7.22 in 2012 (APC: 0.83; 95% CI: −0.17 to 1.85; *p* = 0.10), which was followed by a significant increase from 7.22 in 2012 to 5.34 in 2020 (APC: 6.66*; 95% CI: 4.90–8.45; *p* < 0.001) (Figure 5) (Supplementary Tables 3, 7).

### 3.6 Stratified by age groups

We also analyzed data for age groups: 45–64 and 65+. The highest number of deaths occurred in the 65+ age group (number of deaths: 33,848; overall AAMR: 3.05), followed by the 45–64 age group (number of deaths: 2,853; overall AAMR: 0.14).

The overall trend was stable for adults aged 45–64 as the AAMR increased nonsignificantly from 0.18 to 0.22 between 1999 and 2023 (AAPC: 1.74; 95% CI: −0.75 to 4.30; *p* = 0.17), with notable fluctuations across periods. A significant decrease was observed from 0.18 in 1999 to 0.08 in 2010 (APC: −4.94*; 95% CI: −9.51 to −0.14; *p* = 0.044), followed by a significant increase from 0.08 in 2010 to 0.22 in 2023 (APC: 7.76*; 95% CI: 5.07 to 10.52; *p* = 0.005).

**Figure 6:**
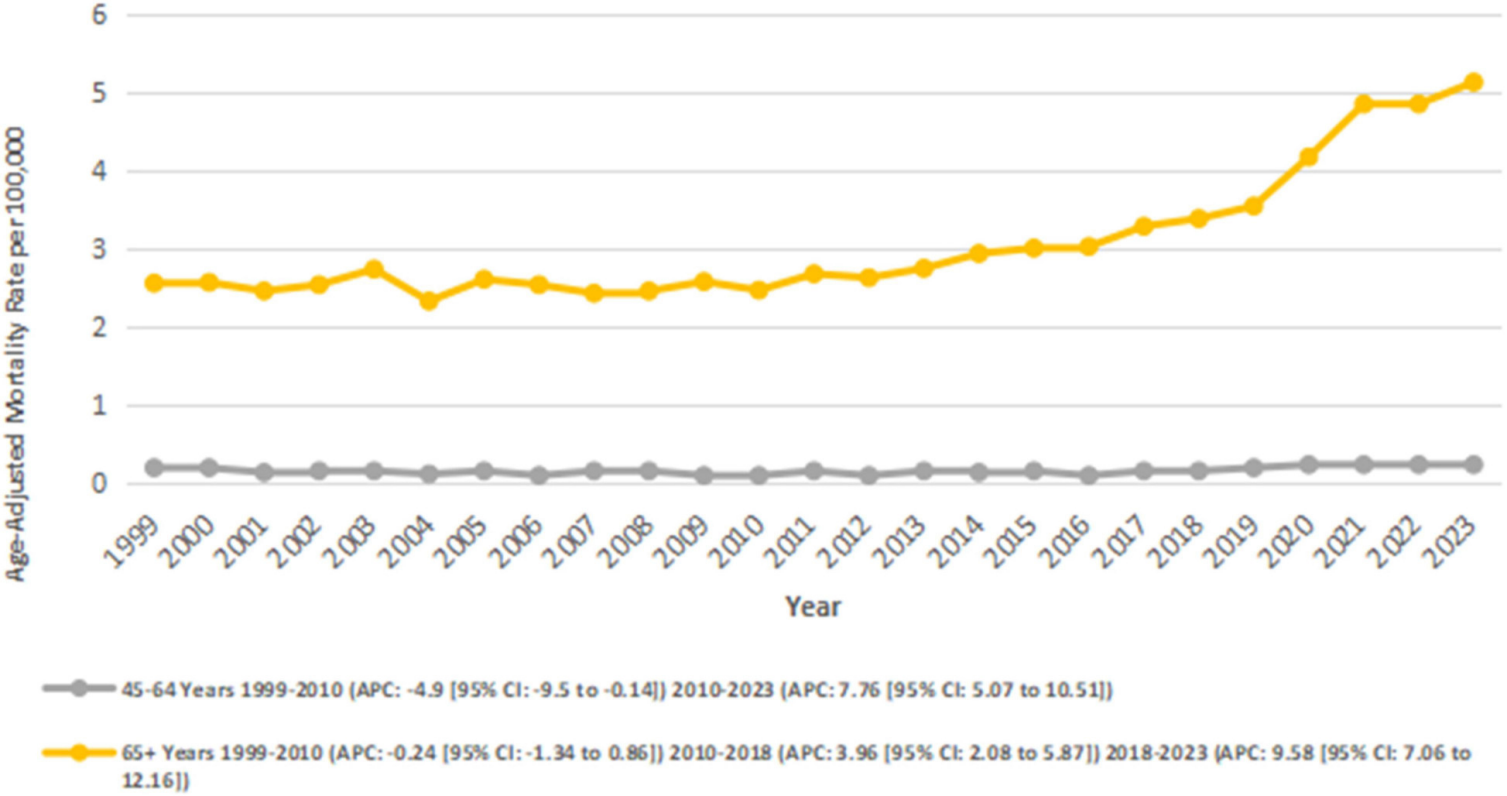
Atrial fibrillation and rheumatic heart disease-related AAMRs per 100,000 stratified by Age in adults in the United States from 1999 to 2023.

In the 65+ age group, there was a significant overall increase in AAMR from 2.55 in 1999 to 5.13 in 2023 (AAPC: 3.14*; 95% CI: 2.25–4.03; *p* < 0.001). This group experienced a mild and nonsignificant decline in AAMRs from 2.55 in 1999 to 2.46 in 2010 (APC: −0.24; 95% CI: −1.34 to 0.87; *p* = 0.65), followed by a significant increase from 2.46 in 2010 to 3.38 in 2018 (APC: 3.96*; 95% CI: 2.09–5.87; *p* = 0.003), and from 3.38 in 2018 to 5.13 in 2023 (APC: 9.59*; 95% CI: 7.07–12.17; *p* < 0.001) (Figure 6) (Supplementary Tables 3, 8).

## 4 Discussion

AF and RHD are of major concern to cardiovascular disease entities globally—especially for low- and middle-income countries but still a latent contributor to morbidity and mortality in higher-income countries such as the United States (12, 23, 24). Together they bring risk and exacerbate negative cardiovascular events by creating a separate, high-risk population. In this study, we present the study of national mortality trends from all cause deaths related to AF-RHD-related deaths across groups, geographic regions, and structural characteristics from 1999 to 2023.

Among US adults ages 45 and older, there were 36,701 deaths attributed to AF-RHD from 1999 to 2023. For adults 45 and older, the AAMR rose dramatically from 1.04 to 2.00. This upward trend was consistent with the national data showing an increasing mortality rate especially among older adults with AF-RHD, most likely due to an increasing burden of comorbidities (25–27). In the first decade, 1999–2010, there was a decline in AAMR, though not statistically significant. We speculate the decline might reflect some small early on cardiovascular improvements due to changing care and a focus on secondary prevention. After 2010, there was a steady and statistically significant increase in AAMR related to AF-RHD with the largest increase from 2018 to 2021 as potentially driven by age, more comorbidities, later complications and delayed chronic care management in the wake of COVID-19 (26, 28). The period from 2021 to 2023 appears to show a disturbing plateau, which could be attributable to an observational lag and/or time lag associated with system recovery, but we will need to continue to monitor the trends.

Women demonstrated a quite disproportionate higher death rate and consistent higher AAMR, despite AF usually predominating in men. This sex-based paradox exists amongst multiple population-based work, possibly because women with AF have greater stroke risk, which may also be related to under-treatment, under-diagnosis (27, 29, 30). The increased mortality was in both sexes post-2010, with recurrently higher relative increase in mortality shown in men compared to women. This recurring increase of death, is consistent with emerging literature regarding sex-differences in AF complications and suggests more nuanced clinical algorithms be developed that incorporate specific female risk stratification (31, 32). Notably, women experienced approximately twice the mortality rate of men, highlighting a substantial and persistent sex-based disparity. This finding reinforces evidence that women with AF often present later, receive less aggressive rhythm or anticoagulation therapy, and may have underrecognized valvular or structural heart disease. The higher mortality among females despite the lower overall prevalence of AF underscores the importance of sex-specific diagnostic vigilance, individualized anticoagulation strategies, and equitable access to specialized cardiovascular care.

We stratified by race and ethnicity and noted considerable differences. NH white individuals had the largest estimated absolute mortality burden and the highest age-adjusted mortality risk. The NH White data showed an interesting trajectory; there was a slight decline until 2010, and then there was a relatively large and sustained increase. This trajectory mirrors wider cardiometabolic literature describing increased mortality in mid-to-later life White Americans attributed to chronic disease and behavioral health comorbidities (33, 34). NH black adults had the most delayed rise in mortality rates, however the recent steep rise may confirm systemic barriers to timely diagnosis and anticoagulation, as well as a higher baseline prevalence of hypertension and heart failure (35). Hispanic adults also showed reversal from early declines to large increases in mortality rates post-2013.

Geographic differences were notable, with the highest overall AAMRs in the West, followed the Midwest, Northeast, and South. The higher mortality in the Western region may be multifactorial, reflecting demographic shifts, aging population growth, limited access to specialized cardiovascular care in rural Western states, and higher prevalence of metabolic risk factors such as obesity and diabetes. These factors collectively contribute to delayed diagnosis, treatment gaps, and poorer outcomes in AF-RHD patients (36, 37). Decreasing rates for the Northeast from 1999 to 2008 also had decent rate increases closely followed in other regions. The South being described as the “Stroke Belt” had the lowest overall baseline AAMRs but had the largest increase post-2017 for all regions reflective of a regional increase in cardiometabolic syndromes and healthcare access challenges (38). The West and Midwest realized significant epidemics in their late mortality AAMR rates. This trend highlights issues of healthcare infrastructure, deferred outpatient management or limited local expertise in forward rheumatic disease follow-up on mortality rates (2, 39). Regional variation supports a rationale for local context specific intervention strategies based on regional epidemiology, health system capacity and social determinants (40).

Urbanization analysis indicated that non-metropolitan areas had greater AAMR than metropolitan areas. Non-metropolitan mortality steadily increased overall, with an overwhelming temporal increase between 2012 and 2020. The fact that the mortality rates in rural areas are higher correlates with other cardiovascular findings that infer a multitude of concepts stemmed from the access to specialists earlier in the disease course, distance travelled to see specialist services, socioeconomic status, and hospital closures (41, 42). In urban metropolitan areas, AAMRs declined strongly in the early period, but the rate of decline slowed significantly after around 2010, consistent with previous studies showing a plateau in urban cardiovascular mortality trends (43).

Mortality was primarily seen in adults aged ≥65, where a clear increase in AAMR from 1999 to 2023. The acceleration since 2018 likely reflects increased frailty, polypharmacy, and the late AF/RHD complications in older patients (44). Adults aged 45–64 had much lower mortality, but also had a faster increase since 2010 which points to earlier onset, or delayed treatment in mid-life. This highlights the pressing need for age-appropriate primary prevention, especially adherence to anticoagulation and early detection of valvular disease in both middle aged and older adults (45, 46).

## 5 Strengths and limitations

This study represents the most recent longitudinal assessment of trends in AF-RHD mortality in the U.S. using comprehensive CDC WONDER data while stratifying by multiple variables. The longitudinal aspect of 25 years, a joinpoint analysis, and potential comparisons based on age-adjusted mortality rates enhances the interpretability and rigor of the findings. Limitations of this assessment include reliance on death certificate data, which may have misdiagnosed or downplayed AF or RHD as a contributory cause of a patient's death.

Also, there was no data collected on socioeconomic status, clinical comorbidities, and treatment exposures, which limits causal inference. Additionally, some racial categories and suppression of certain state-level data made it impossible to conduct some analysis.

## 6 Clinical and policy implications

The findings point to an urgent need for integrated management of AF and RHD, especially among women, older people, and populations outside of metropolitan areas. Population health initiatives ought to advocate for increased use of screening for valvular dysfunction, enhanced access to anticoagulation clinics, and expanded telehealth options for rural living (47–49). Geographic and racial disparities considered, explicit consideration of health inequities should inform policy changes that enable integrated management, culturally competent care access, accessibility to cardiac rehabilitation programs, and prevention initiatives built within communities (50–53). In light of the dramatic increase in mortality that began after 2018, and the dramatic rise in AF-RHD multifactorial approaches to improving healthcare system readiness for crises (such as pandemics) is warranted.

## 7 Conclusion and future directions

Between 1999 and 2023, mortality due to AF and RHD almost doubled in the United States, with the most pronounced acceleration occurring over the past decade. Substantial disparities by sex, race, region, and urbanization persist and appear to be widening. These trends highlight the need for prevention, early detection and equitable access to specialized cardiovascular care. Future studies designed to investigate these mortality trends should use real-world longitudinal cohorts and evaluate the impact of health system interventions to mitigate the mortality trends related to atrial fibrillation and rheumatic heart disease.

## Data Availability

Publicly available datasets were used in this study. The data can be accessed through the CDC WONDER platform, including the Multiple Cause of Death database available at: https://wonder.cdc.gov/mcd.html.
