# Supplementary material for: Temporal trends in mortality involving atrial fibrillation and rheumatic heart disease: a 25-year nationwide analysis
Source: Front Cardiovasc Med. 2025 Dec 12;12:1687555. doi: 10.3389/fcvm.2025.1687555 (PMC12741100; doi:10.3389/fcvm.2025.1687555)
Supplement: Supplementary file 1 [file Datasheet1.pdf]

Supplemental Table 1 Atrial Fibrillation and Rheumatic Heart Disease–related Deaths, Stratified by Sex and Race, in Adults in the United States, 1999 to 2023

Year	Overall	Women	Men	NH White	NH Black	Hispanic/Latino
1999	989	707	282	886	39	36
2000	986	703	283	906	33	28
2001	967	699	268	865	38	39
2002	997	706	291	898	37	29
2003	1,071	726	345	976	42	29
2004	904	635	269	796	40	34
2005	1,047	730	317	938	33	41
2006	1,040	729	311	936	37	42
2007	1,027	723	304	920	44	35
2008	1,071	724	347	962	42	37
2009	1,121	737	384	978	48	57
2010	1,077	728	349	959	39	34
2011	1,214	812	402	1,080	56	47
2012	1,241	808	433	1,107	52	47
2013	1,332	864	468	1,172	57	47
2014	1,440	943	497	1,240	72	73
2015	1,510	1,016	494	1,322	67	76
2016	1,546	1,013	533	1,364	62	65
2017	1,723	1,144	579	1,496	81	86
2018	1,838	1,187	651	1,578	97	89
2019	1,965	1,272	693	1,686	107	86
2020	2,368	1,509	859	2,007	148	109
2021	2,587	1,607	980	2,193	151	140
2022	2,786	1,772	1,014	2,383	160	138
2023	2,854	1,780	1,074	2,407	172	150
**Total**	36,701	24,274	12,427	32,055	1,754	1,594

NH, non-Hispanic

Supplemental Table 2 Atrial Fibrillation and Rheumatic Heart Disease –related Mortality, Stratified by Place of Death in Adults in the United States, 1999 to 2023

**Place of Death**	**Deaths**
Medical Facility - Inpatient	13,939
Medical Facility - Outpatient or ER	1,666
Medical Facility - Dead on Arrival	103
Medical Facility - Status unknown	12
Decedent's home	11,019
Hospice facility	1,847
Nursing home/long term care	6,530
Other	1,537
Place of death unknown	40
**Total**	36,693

Supplemental Table 3 Annual percent change (APC) of Atrial Fibrillation and Rheumatic Heart Disease –related Age-Adjusted Mortality Rates per 100,000 in Adults in the United States, 1999 to 2023

Year	APC (95% CI)
**Overall**	
1999-2010	−0.56 (−1.4 to 0.35)
2010-2018	3.68 (2.03 to 5.36)
2018-2021	12.94 (3.17 to 23.6)
2021-2023	3.31 (−5.9 to 13.4)
**Women**	
1999-2010	−1.33 (−2.35 to −0.3)
2010-2018	4.17 (2.39 to 5.99)
2018-2023	8.53 (6.09 to 11.02)
**Men**	
1999-2016	1.71 (0.75 to 2.67)
2016-2023	10.29 (7.46 to 13.19)
**NH White**	
1999-2010	−0.38 (−1.6 to 0.83)
2010-2017	4.13 (1.36 to 6.98)
2017-2023	9.13 (6.71 to 11.6)
**NH Black**	
1999-2016	1.83 (0.61 to 3.07)
2016-2023	12.6 (9.4 to 15.8)

**Hispanic/Latino**	
1999-2013	−1.04 (−3.18 to 1.14)
2013-2023	6.81 (4.51 to 9.15)
**Metropolitan Areas**	
1999-2010	−1.1 (−2.48 to 0.11)
2010-2020	4.57 (3.37 to 5.78)
**Non-metropolitan Areas**	
1999-2012	0.83 (−0.17 to 1.84)
2012-2020	6.6 (4.89 to 8.44)

APC = annual percent change; NH = non-Hispanic.

Supplemental Table 4 Overall and Sex-Stratified Atrial Fibrillation and Rheumatic Heart Disease –related Age-Adjusted Mortality Rates per 100,000 in Adults in the United States, 1999 to 2023.

Year	Overall	Women	Men
1999	1.04 (0.97-1.1)	1.21 (1.12-1.3)	0.8 (0.7-0.89)
2000	1.04 (0.98-1.11)	1.2 (1.11-1.29)	0.76 (0.66-0.85)
2001	0.97 (0.9-1.03)	1.17 (1.08-1.25)	0.72 (0.63-0.81)
2002	1.01 (0.94-1.07)	1.19 (1.1-1.28)	0.77 (0.68-0.86)
2003	1.08 (1.01-1.14)	1.18 (1.09-1.26)	0.91 (0.81-1)
2004	0.9 (0.84-0.96)	1.03 (0.95-1.12)	0.67 (0.59-0.75)
2005	1.03 (0.97-1.09)	1.15 (1.07-1.24)	0.8 (0.71-0.89)
2006	0.97 (0.91-1.03)	1.14 (1.06-1.22)	0.74 (0.66-0.83)
2007	0.97 (0.91-1.03)	1.11 (1.03-1.19)	0.74 (0.66-0.83)
2008	0.98 (0.92-1.03)	1.08 (1-1.16)	0.83 (0.74-0.92)
2009	0.98 (0.93-1.04)	1.05 (0.97-1.13)	0.87 (0.78-0.96)
2010	0.94 (0.89-1)	1.04 (0.96-1.11)	0.75 (0.67-0.83)
2011	1.06 (1-1.12)	1.14 (1.06-1.22)	0.86 (0.77-0.94)
2012	1 (0.94-1.06)	1.07 (1-1.15)	0.89 (0.81-0.98)
2013	1.08 (1.02-1.14)	1.18 (1.1-1.26)	0.97 (0.88-1.06)
2014	1.14 (1.08-1.2)	1.24 (1.16-1.32)	0.95 (0.87-1.04)
2015	1.18 (1.12-1.24)	1.3 (1.22-1.39)	0.96 (0.87-1.05)
2016	1.15 (1.09-1.2)	1.28 (1.2-1.36)	0.99 (0.91-1.08)
2017	1.28 (1.22-1.34)	1.41 (1.32-1.49)	1.07 (0.98-1.15)
2018	1.32 (1.25-1.38)	1.45 (1.36-1.53)	1.15 (1.06-1.24)
2019	1.4 (1.33-1.46)	1.51 (1.42-1.59)	1.17 (1.08-1.26)
2020	1.65 (1.59-1.72)	1.78 (1.69-1.87)	1.41 (1.32-1.51)
2021	1.9 (1.82-1.97)	1.99 (1.89-2.09)	1.78 (1.67-1.89)
2022	1.9 (1.83-1.97)	2.03 (1.94-2.13)	1.71 (1.6-1.82)
2023	2 (1.92-2.07)	2.09 (1.99-2.19)	1.8 (1.69-1.91)

Supplemental Table 5 Atrial Fibrillation and Rheumatic Heart Disease –related Age-Adjusted Mortality Rates per 100,000, Stratified by Race in Adults in the United States, 1999 to 2023

Year	NH White	NH Black	Hispanic
1999	1.09 (1.02-1.17)	0.47 (0.33-0.65)	0.71 (0.49-1)
2000	1.1 (1.02-1.17)	0.39 (0.27-0.56)	0.57 (0.38-0.83)
2001	1.03 (0.96-1.09)	0.48 (0.34-0.67)	0.72 (0.5-1)
2002	1.08 (1.01-1.15)	0.45 (0.32-0.63)	0.58 (0.38-0.83)
2003	1.18 (1.1-1.25)	0.49 (0.35-0.67)	0.54 (0.36-0.78)
2004	0.91 (0.85-0.98)	0.47 (0.34-0.65)	0.6 (0.41-0.85)
2005	1.06 (0.99-1.13)	0.39 (0.27-0.55)	0.67 (0.47-0.91)
2006	1.06 (1-1.13)	0.42 (0.29-0.58)	0.7 (0.5-0.96)
2007	1.05 (0.98-1.11)	0.46 (0.33-0.63)	0.51 (0.34-0.72)
2008	1.03 (0.97-1.1)	0.46 (0.33-0.62)	0.52 (0.36-0.72)
2009	1.06 (0.99-1.12)	0.46 (0.34-0.62)	0.77 (0.58-1.01)
2010	1 (0.93-1.06)	0.36 (0.25-0.5)	0.48 (0.33-0.68)
2011	1.11 (1.04-1.18)	0.54 (0.4-0.7)	0.59 (0.43-0.79)
2012	1.11 (1.05-1.18)	0.52 (0.39-0.69)	0.54 (0.39-0.73)
2013	1.21 (1.14-1.28)	0.5 (0.37-0.65)	0.48 (0.35-0.65)
2014	1.2 (1.13-1.27)	0.63 (0.49-0.8)	0.69 (0.53-0.88)
2015	1.3 (1.23-1.37)	0.56 (0.43-0.71)	0.75 (0.59-0.95)
2016	1.28 (1.21-1.35)	0.51 (0.39-0.66)	0.63 (0.48-0.81)
2017	1.4 (1.32-1.47)	0.63 (0.49-0.78)	0.77 (0.61-0.96)
2018	1.47 (1.4-1.54)	0.72 (0.58-0.88)	0.75 (0.6-0.93)
2019	1.54 (1.47-1.62)	0.79 (0.63-0.94)	0.71 (0.56-0.88)
2020	1.79 (1.72-1.87)	1.03 (0.86-1.2)	0.82 (0.66-0.98)
2021	2.11 (2.02-2.2)	1.05 (0.88-1.23)	1.07 (0.89-1.26)
2022	2.15 (2.07-2.24)	1.14 (0.96-1.33)	1.03 (0.85-1.2)
2023	2.21 (2.12-2.29)	1.2 (1.02-1.39)	1.1 (0.92-1.28)

NH = non-Hispanic.

Supplemental Table 6 Atrial Fibrillation and Rheumatic Heart Disease –related Age-Adjusted Mortality Rates per 100,000, Stratified by Census Region in Adults in the United States, 1999 to 2023.

Census Region	Year	Age-Adjusted Mortality Rate (95% CI)
Northeast	1999	1.19 (1.04-1.34)
Northeast	2000	1.11 (0.97-1.26)
Northeast	2001	1.09 (0.95-1.24)
Northeast	2002	0.98 (0.84-1.11)
Northeast	2003	1.13 (0.98-1.27)
Northeast	2004	0.93 (0.8-1.06)
Northeast	2005	0.94 (0.81-1.07)
Northeast	2006	0.88 (0.76-1.01)
Northeast	2007	0.85 (0.73-0.97)
Northeast	2008	0.81 (0.69-0.93)
Northeast	2009	0.82 (0.71-0.94)
Northeast	2010	0.99 (0.86-1.12)
Northeast	2011	0.91 (0.79-1.03)
Northeast	2012	1.02 (0.89-1.15)
Northeast	2013	1.1 (0.96-1.23)
Northeast	2014	1.25 (1.11-1.39)
Northeast	2015	1.12 (0.98-1.25)
Northeast	2016	1.04 (0.91-1.17)
Northeast	2017	1.18 (1.04-1.31)
Northeast	2018	1.2 (1.07-1.34)
Northeast	2019	1.23 (1.1-1.36)
Northeast	2020	1.67 (1.51-1.83)
Northeast	2021	1.63 (1.48-1.79)
Northeast	2022	1.75 (1.59-1.91)
Northeast	2023	1.84 (1.68-2)

Midwest	1999	1.04 (0.91-1.18)
Midwest	2000	1.13 (0.99-1.27)
Midwest	2001	0.88 (0.76-1.01)
Midwest	2002	1.22 (1.08-1.36)
Midwest	2003	1.11 (0.97-1.24)
Midwest	2004	0.92 (0.8-1.04)
Midwest	2005	1.16 (1.03-1.3)
Midwest	2006	1.06 (0.94-1.19)
Midwest	2007	1.17 (1.03-1.31)
Midwest	2008	1.28 (1.14-1.42)
Midwest	2009	1.16 (1.03-1.29)
Midwest	2010	0.99 (0.87-1.11)
Midwest	2011	1.16 (1.03-1.29)
Midwest	2012	1.15 (1.02-1.28)
Midwest	2013	1.17 (1.04-1.3)
Midwest	2014	1.18 (1.05-1.3)
Midwest	2015	1.42 (1.27-1.56)
Midwest	2016	1.46 (1.32-1.6)
Midwest	2017	1.57 (1.42-1.71)
Midwest	2018	1.61 (1.47-1.76)
Midwest	2019	1.61 (1.47-1.76)
Midwest	2020	1.93 (1.77-2.08)
Midwest	2021	2.09 (1.92-2.25)
Midwest	2022	1.99 (1.83-2.15)
Midwest	2023	2.2 (2.03-2.37)
South	1999	0.75 (0.66-0.85)
South	2000	0.75 (0.66-0.85)
South	2001	0.82 (0.72-0.92)
South	2002	0.73 (0.64-0.82)
South	2003	0.84 (0.74-0.94)
South	2004	0.62 (0.54-0.7)

South	2005	0.69 (0.61-0.78)
South	2006	0.68 (0.59-0.76)
South	2007	0.67 (0.59-0.75)
South	2008	0.65 (0.57-0.73)
South	2009	0.7 (0.61-0.78)
South	2010	0.65 (0.57-0.73)
South	2011	0.8 (0.71-0.88)
South	2012	0.73 (0.65-0.81)
South	2013	0.79 (0.7-0.87)
South	2014	0.86 (0.78-0.95)
South	2015	0.85 (0.77-0.94)
South	2016	0.77 (0.69-0.84)
South	2017	0.76 (0.69-0.84)
South	2018	0.92 (0.83-1)
South	2019	1 (0.91-1.08)
South	2020	1.1 (1.01-1.19)
South	2021	1.43 (1.32-1.53)
South	2022	1.49 (1.39-1.6)
South	2023	1.54 (1.44-1.65)
West	1999	1.37 (1.2-1.53)
West	2000	1.26 (1.1-1.42)
West	2001	1.31 (1.15-1.48)
West	2002	1.22 (1.07-1.38)
West	2003	1.41 (1.25-1.58)
West	2004	1.25 (1.09-1.4)
West	2005	1.36 (1.2-1.52)
West	2006	1.46 (1.29-1.62)
West	2007	1.3 (1.15-1.45)
West	2008	1.31 (1.16-1.46)
West	2009	1.42 (1.27-1.57)
West	2010	1.28 (1.14-1.43)

West	2011	1.38 (1.23-1.53)
West	2012	1.35 (1.21-1.5)
West	2013	1.46 (1.31-1.6)
West	2014	1.48 (1.34-1.63)
West	2015	1.51 (1.36-1.65)
West	2016	1.62 (1.47-1.77)
West	2017	1.83 (1.68-1.99)
West	2018	1.81 (1.66-1.96)
West	2019	1.94 (1.79-2.1)
West	2020	2.24 (2.08-2.41)
West	2021	2.68 (2.49-2.87)
West	2022	2.67 (2.49-2.85)
West	2023	2.65 (2.47-2.83)

Supplemental Table 7 Atrial Fibrillation and Rheumatic Heart Disease –related Age-Adjusted Mortality Rates per 100,000, Stratified by Urban-Rural Classification in Adults in the United States, 1999 to 2020.

Year	Metropolitan	Non-metropolitan
1999	1.07 (0.99-1.14)	0.92 (0.78-1.06)
2000	1.04 (0.97-1.12)	1.02 (0.87-1.17)
2001	1 (0.93-1.07)	0.91 (0.78-1.05)
2002	1.03 (0.96-1.1)	0.96 (0.82-1.1)
2003	1.09 (1.02-1.17)	1.1 (0.94-1.25)
2004	0.88 (0.81-0.94)	0.97 (0.83-1.11)
2005	1.03 (0.96-1.1)	1.02 (0.87-1.16)
2006	0.95 (0.88-1.01)	1.05 (0.91-1.19)
2007	0.95 (0.89-1.02)	0.94 (0.8-1.08)
2008	0.98 (0.92-1.05)	1.01 (0.87-1.16)
2009	0.94 (0.88-1.01)	1.14 (0.99-1.29)
2010	0.91 (0.85-0.97)	0.96 (0.83-1.1)
2011	1.05 (0.99-1.12)	1.05 (0.91-1.19)
2012	0.98 (0.92-1.05)	1.07 (0.93-1.21)
2013	1.07 (1.01-1.14)	1.21 (1.06-1.36)
2014	1.09 (1.02-1.15)	1.24 (1.09-1.39)
2015	1.15 (1.08-1.22)	1.31 (1.16-1.46)
2016	1.12 (1.06-1.19)	1.29 (1.14-1.44)
2017	1.24 (1.18-1.31)	1.45 (1.29-1.61)
2018	1.27 (1.21-1.34)	1.61 (1.44-1.78)
2019	1.36 (1.29-1.43)	1.58 (1.42-1.74)
2020	1.6 (1.53-1.67)	1.88 (1.7-2.05)

Supplemental Table 8 Atrial Fibrillation and Rheumatic Heart Disease –related Age-Adjusted Mortality Rates per 100,000, Stratified by Age in Adults in the United States, 1999 to 2023.

Year	45-64 Years	65+ Years
1999	0.18 (0.15-0.21)	2.55 (2.38-2.72)
2000	0.18 (0.14-0.22)	2.56 (2.39-2.73)
2001	0.12 (0.09-0.15)	2.45 (2.29-2.62)
2002	0.14 (0.11-0.17)	2.53 (2.37-2.69)
2003	0.14 (0.11-0.17)	2.73 (2.56-2.9)
2004	0.1 (0.08-0.13)	2.32 (2.16-2.47)
2005	0.14 (0.11-0.18)	2.6 (2.43-2.76)
2006	0.08 (0.06-0.1)	2.53 (2.37-2.69)
2007	0.14 (0.11-0.17)	2.42 (2.27-2.58)
2008	0.14 (0.11-0.17)	2.45 (2.29-2.6)
2009	0.08 (0.06-0.1)	2.57 (2.42-2.73)
2010	0.08 (0.06-0.1)	2.46 (2.31-2.62)
2011	0.14 (0.11-0.17)	2.67 (2.51-2.83)
2012	0.08 (0.06-0.1)	2.62 (2.47-2.77)
2013	0.14 (0.11-0.17)	2.74 (2.59-2.89)
2014	0.12 (0.09-0.14)	2.93 (2.77-3.08)
2015	0.14 (0.11-0.17)	3 (2.84-3.15)
2016	0.08 (0.06-0.09)	3.02 (2.86-3.18)
2017	0.14 (0.11-0.17)	3.28 (3.12-3.44)
2018	0.14 (0.11-0.17)	3.38 (3.22-3.54)
2019	0.18 (0.15-0.21)	3.54 (3.38-3.7)
2020	0.22 (0.19-0.25)	4.17 (4-4.35)
2021	0.22 (0.19-0.25)	4.85 (4.65-5.04)
2022	0.22 (0.19-0.25)	4.85 (4.66-5.03)
2023	0.22 (0.19-0.25)	5.13 (4.93-5.32)
